# Supplementary material for: SARS-CoV-2 nsp15 preferentially degrades AU-rich dsRNA via its dsRNA nickase activity
Source: Nucleic Acids Res. 2024 Apr 18;52(9):5257–72. doi: 10.1093/nar/gkae290 (PMC11109939; doi:10.1093/nar/gkae290)
Supplement: gkae290_Supplemental_Files [file gkae290_supplemental_files.zip › Supplementary Figures.pdf]

Supplementary Material

SARS-CoV-2 nsp15 preferentially degrades AU-rich dsRNA via its dsRNA nickase activity

Xionglue Wang[1] and Bin Zhu[1,2,*]

[1] Key Laboratory of Molecular Biophysics, the Ministry of Education, College of Life Science and Technology, Huazhong University of Science and Technology, Wuhan, Hubei 430074, China

[2] Shenzhen Huazhong University of Science and Technology Research Institute, Shenzhen 518063, China

[*] To whom correspondence should be addressed. Tel: +86 18154323093; Email: bin_zhu@hust.edu.cn

This file contains Supplementary Figures S1-S6. Supplementary Tables S1-S4 are provided in an Excel workbook.

Supplementary Tables:

Table S1. Plasmids and primers for protein expression.

Table S2. Plasmids and primers for IVT DNA templates.

Table S3. RNA substrates synthesized by IVT in this study.

Table S4. AU content of coronavirus genomes.

Figure S1

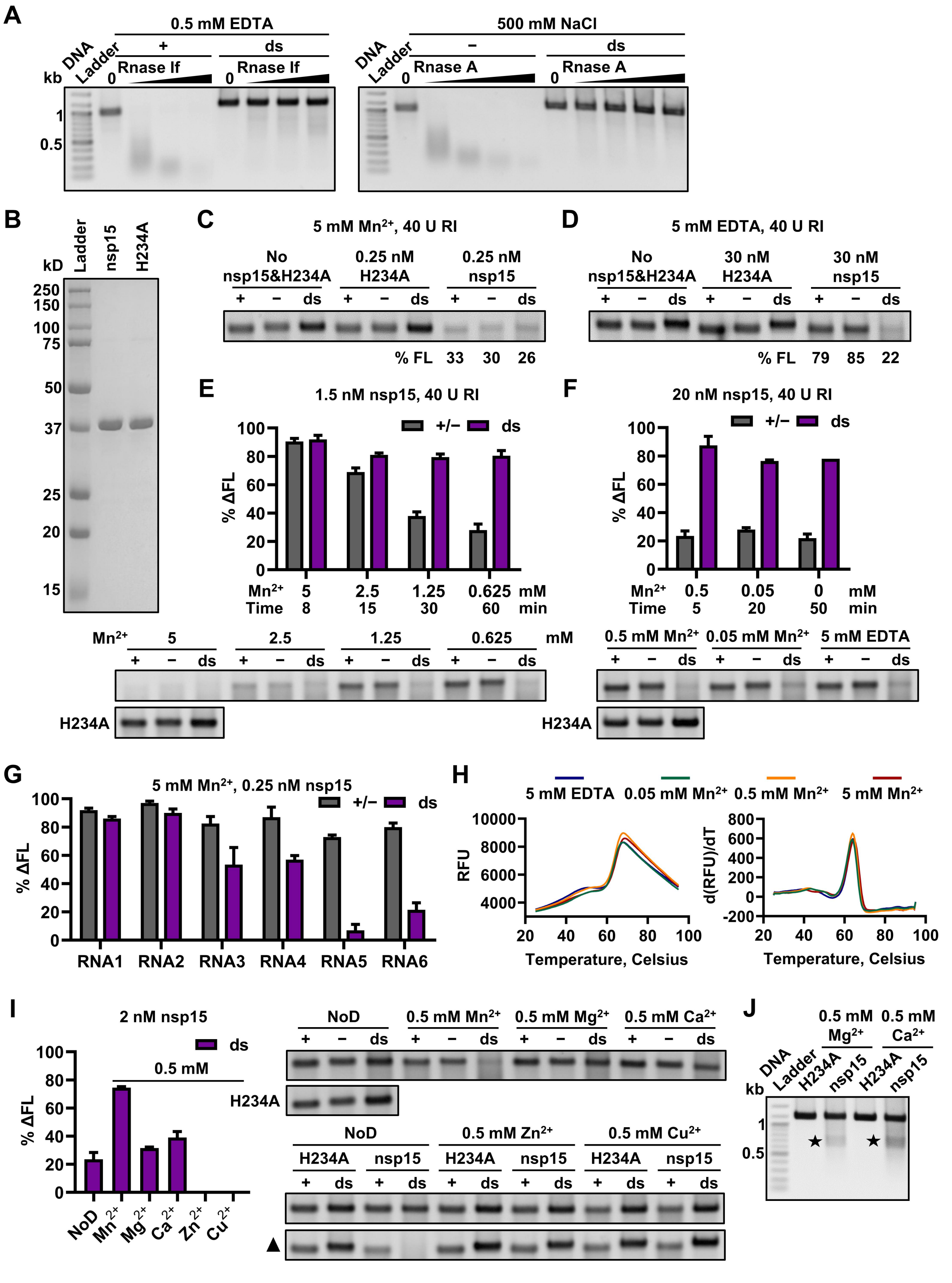
A
0.5 mM EDTA
+
ds
DNA Ladder
kb
0
Rnase If
1
0.5
500 mM NaCl
–
ds
Rnase A
B
Ladder
nsp15
H234A
kD
250
150
100
75
50
37
25
20
15
C
5 mM Mn2+, 40 U RI
No nsp15&H234A
0.25 nM H234A
0.25 nM nsp15
% FL 33 30 26
D
5 mM EDTA, 40 U RI
No nsp15&H234A
30 nM H234A
30 nM nsp15
% FL 79 85 22
E
1.5 nM nsp15, 40 U RI
+/– ds
% ΔFL
Mn2+ 5 2.5 1.25 0.625 mM
Time 8 15 30 60 min
Mn2+ 5 2.5 1.25 0.625 mM
H234A
F
20 nM nsp15, 40 U RI
Mn2+ 0.5 0.05 0 mM
Time 5 20 50 min
0.5 mM Mn2+
0.05 mM Mn2+
5 mM EDTA
H234A
G
5 mM Mn2+, 0.25 nM nsp15
RNA1 RNA2 RNA3 RNA4 RNA5 RNA6
H
5 mM EDTA
0.05 mM Mn2+
0.5 mM Mn2+
5 mM Mn2+
RFU
d(RFU)/dT
Temperature, Celsius
I
2 nM nsp15
ds
0.5 mM
NoD Mn2+ Mg2+ Ca2+ Zn2+ Cu2+
NoD
0.5 mM Mn2+
0.5 mM Mg2+
0.5 mM Ca2+
H234A
0.5 mM Zn2+
0.5 mM Cu2+
nsp15
J
0.5 mM Mg2+
0.5 mM Ca2+
DNA Ladder
H234A
nsp15
kb
1
0.5

Figure S1. Effect of Mn^{2+} and other divalent metal ions on the cleavage activity of nsp15 (related to Figure 1).

(**A**) Verification of the 1-kb annealing product as double-stranded RNA (dsRNA) using RNase I and RNase A. (**B**) SDS-PAGE analysis of purified wild-type SARS-CoV-2 nsp15 (approximately 40 kDa including an N-terminal 6× His tag) and its active-site mutant, H234A. (**C**) Cleavage of the ssRNA and dsRNA substrates by nsp15 in the presence of 5 mM Mn^{2+} and 40 U of murine RNase inhibitor (RI). The full gel image is shown in **Supplementary Figure S6B**. (**D**) Cleavage of the ssRNA and dsRNA substrates by nsp15 in the presence of 5 mM EDTA and 40 U of RI. The full gel image is shown in **Supplementary Figure S6C**. In (**C and D**), remaining full-length RNA substrates after nsp15 cleavage were quantified as % FL and the nsp15 H234A mutant was used as a negative control. (**E**) Cleavage of the ssRNA and dsRNA substrates by nsp15 at various Mn^{2+} concentrations (>0.5 mM) and reaction times. The full gel image is shown in **Supplementary Figure S6D**. (**F**) Cleavage of the ssRNA and dsRNA substrates by nsp15 at various Mn^{2+} concentrations (≤0.5 mM) and reaction times. The full gel image is shown in **Supplementary Figure S6E**. (**G**) Cleavage of RNA substrates 1–6 (as shown in **Figure 1E**) by nsp15 in the presence of 5 mM Mn^{2+}. The full gel images are shown in **Supplementary Figure S6F**. (**H**) Differential scanning fluorimetry results for nsp15 in the presence of varying concentrations of Mn^{2+}. The first derivative of the melt curve (as shown on the left) produced a peak (as shown on the right), which provided the melting temperature (T_m). RFU refers to relative fluorescence unit. (**I**) Cleavage of the ssRNA and dsRNA substrates by nsp15 in the presence of various divalent metal ions of 0.5 mM concentration. NoD refers to no ion control. The black triangle indicates increasing the concentration of nsp15 to 30 nM. The full gel images are shown in **Supplementary Figure S6G**. In (**E–G and I**), reduction of the full-length RNA substrates by nsp15 cleavage was quantified as % ΔFL and the nsp15 H234A mutant was used as a negative control. The average and standard deviation for at least two independent reactions are graphed. (**J**) Cleavage of the dsRNA substrates by nsp15 in the presence of 0.5 mM Mg^{2+} or Ca^{2+}. The prominent gel bands indicating specific cleavage are marked by black pentagrams. All RNA substrates were derived from the SARS-CoV-2 mini-genome (as shown in **Figure 1A**) unless otherwise indicated. All samples were analyzed by 1% TAE agarose gel electrophoresis (AGE) (native gel) unless otherwise indicated.

Figure S2A–F

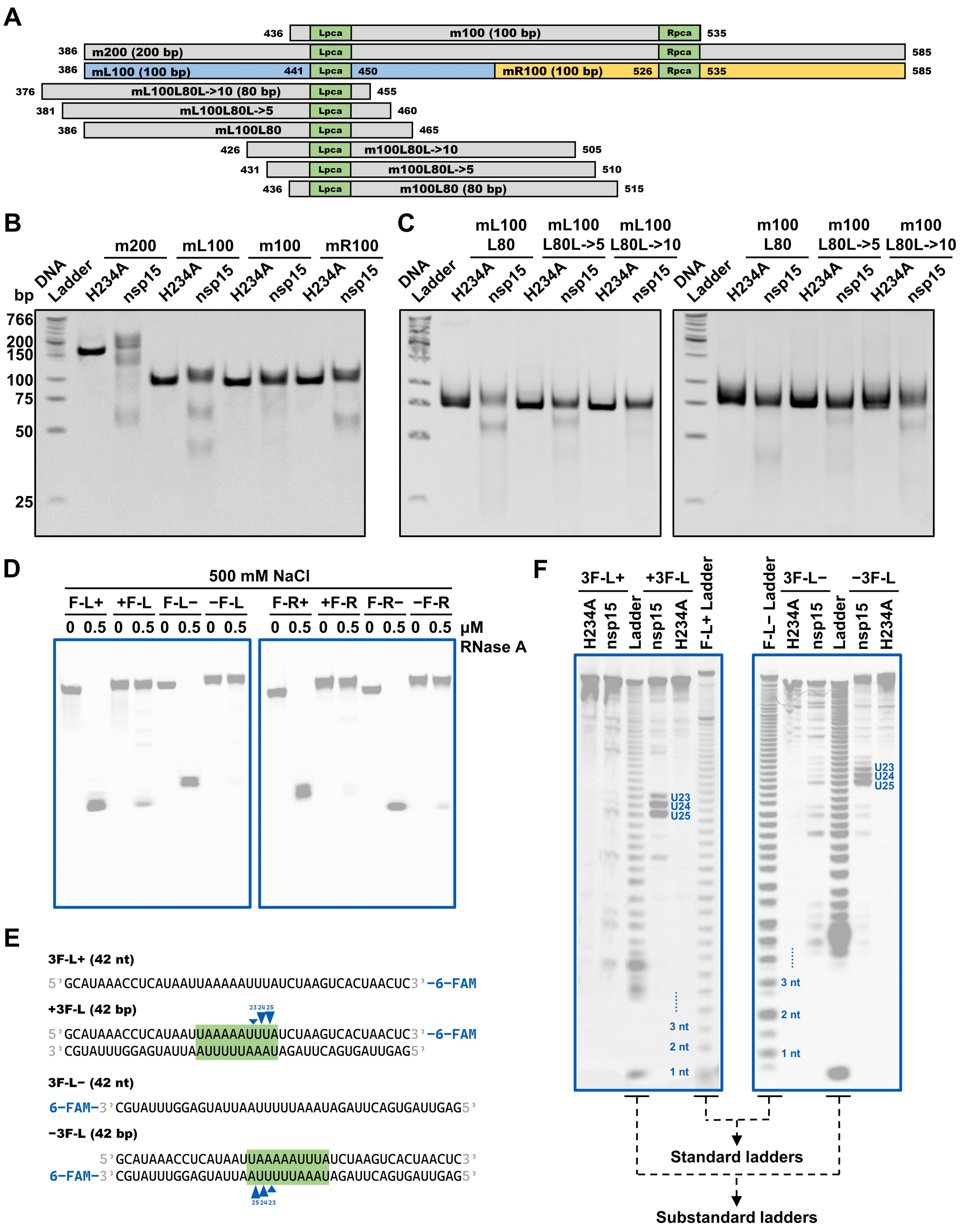

A
m100 (100 bp)
m200 (200 bp)
mL100 (100 bp)
mR100 (100 bp)
mL100L80L->10 (80 bp)
mL100L80L->5
mL100L80
m100L80L->10
m100L80L->5
m100L80 (80 bp)
Lpca
Rpca
B
C
DNA Ladder
H234A
nsp15
D
500 mM NaCl
F-L+
+F-L
F-L−
−F-L
F-R+
+F-R
F-R−
−F-R
μM RNase A
E
3F-L+ (42 nt)
5ʼGCAUAAACCUCAUAAUUAAAAAAUUUAUCUAAGUCACUAACUC3ʼ-6-FAM
+3F-L (42 bp)
5ʼGCAUAAACCUCAUAAUUAAAAAAUUUAUCUAAGUCACUAACUC3ʼ-6-FAM
3ʼCGUAUUUGGAGUAUUAAUUUUUUAAAUAGAUUCAGUGAUUGAG5ʼ
3F-L− (42 nt)
6-FAM-3ʼCGUAUUUGGAGUAUUAAUUUUUUAAAUAGAUUCAGUGAUUGAG5ʼ
−3F-L (42 bp)
5ʼGCAUAAACCUCAUAAUUAAAAAAUUUAUCUAAGUCACUAACUC3ʼ
6-FAM-3ʼCGUAUUUGGAGUAUUAAUUUUUUAAAUAGAUUCAGUGAUUGAG5ʼ
F
3F-L+
+3F-L
3F-L−
−3F-L
F-L+ Ladder
F-L− Ladder
Ladder
U23
U24
U25
3 nt
2 nt
1 nt
Standard ladders
Substandard ladders

Figure S2G, H

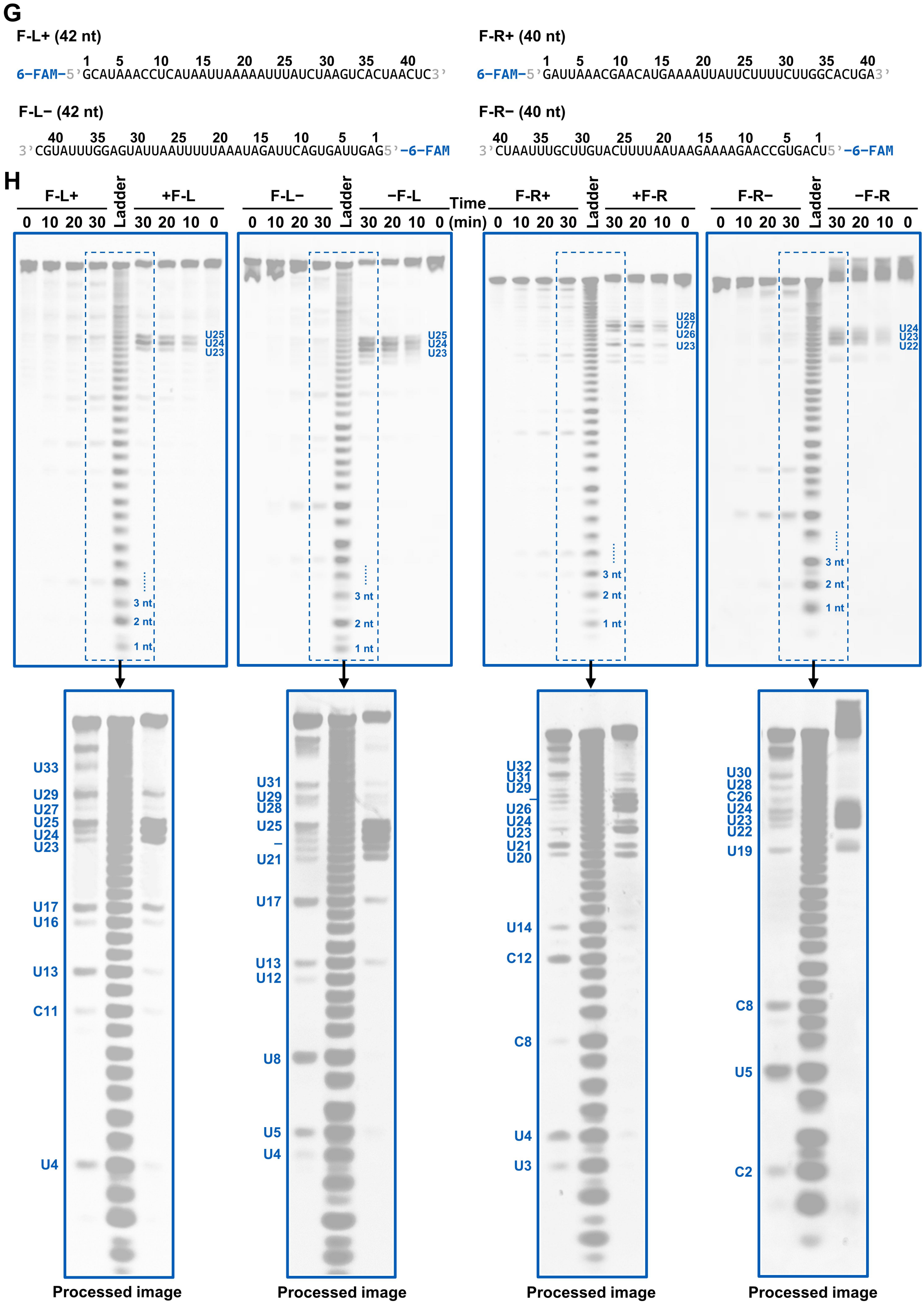
G
F-L+ (42 nt)
6-FAM-5'GCAUAAACCUCAUAAUUAAAAAAUUUAUCUAAGUCACUAACUC3'
F-L− (42 nt)
3'CGUAUUUGGAGUAUUAAUUUUUAAAUAGAUUCAGUGAUUGAG5'-6-FAM
F-R+ (40 nt)
6-FAM-5'GAUUAAACGAACAUGAAAAUUAUUCUUUUCUUGGCACUGA3'
F-R− (40 nt)
3'CUAAUUUGCUUGUACUUUUAAUAAGAAAAGAACCGUGACU5'-6-FAM
H
F-L+
Ladder
+F-L
F-L−
−F-L
Time (min)
F-R+
+F-R
F-R−
−F-R
0 10 20 30
30 20 10 0
U25 U24 U23
U28 U27 U26 U23
U24 U23 U22
3 nt
2 nt
1 nt
U33 U29 U27 U25 U24 U23 U17 U16 U13 C11 U4
U31 U29 U28 U25 U21 U17 U13 U12 U8 U5 U4
U32 U31 U29 U26 U24 U23 U21 U20 U14 C12 C8 U4 U3
U30 U28 C26 U24 U23 U22 U19 C8 U5 C2
Processed image

Figure S2. Cleavage of various dsRNA and ssRNA substrates by nsp15 (related to Figure 2).

(**A**) Schematic showing the dsRNA substrates used in (**B**) and (**C**). (**B**) Locating the preferred cleavage sites of nsp15 in both 386–485-bp and 486–585-bp regions of the O967 substrate. The cleavage products of the m200, mL100, and mR100 substrates, but not the m100 substrate, formed specific gel bands, indicating that nsp15 preferentially cleaved certain sites in the m200, mL100, and mR100 substrates, but not in the m100 substrate. (**C**) Cleavage of nsp15 on dsRNA containing Lpca at various locations. The cleavage products from the mL100L80, mL100L80L->5, m100L80L->10, and m100L80L->5 substrates but not the mL100L80->10 and m100L80 substrates formed specific gel bands corresponding to cleavages in Lpca, suggesting that nsp15 did not cleave Lpca in the mL100L80->10 and m100L80 substrates. In (**B and C**), samples were analyzed by 12% TBE PAGE (native gel). (**D**) Confirmation of the absence of single-stranded regions in the indicated dsRNA substrates using RNase A. (**E**) Schematic representation of the short Lpca-containing dsRNA substrates labeled with 6-FAM at the 3' terminus and the corresponding 6-FAM-labeled ssRNA substrates. The three sites with the strongest cleavage in every strand of the dsRNA substrates are marked with blue triangles, and the cleavage efficiency is indicated by the height of the triangle. (**F**) Identification of the cleavage sites of nsp15 in the RNA substrates shown in (**E**). The alkaline hydrolysis products of the corresponding 3'-labeled and 5'-labeled ssRNA substrates were used as ladders. The three strongest sites in every dsRNA substrate are noted. (**G**) Schematic representation of the 6-FAM-labeled ssRNA substrates corresponding to the Lpca-containing or Rpca-containing dsRNA substrates labeled with 6-FAM in **Figure 2A**. (**H**) Identification of the cleavage sites of nsp15 in the short Lpca-containing or Rpca-containing RNA substrates shown in (**G**) and **Figure 2A**. The three or four strongest cleavage sites in every dsRNA substrate and most distinguishable cleavage sites in every ssRNA substrate are noted. The brightness and contrast of the dashed box areas were enhanced to identify the ssRNA sites that were weakly cleaved by nsp15. In (**D, F, and H**), samples were analyzed by 20% TBE-urea PAGE (denaturing gel).

Figure S3

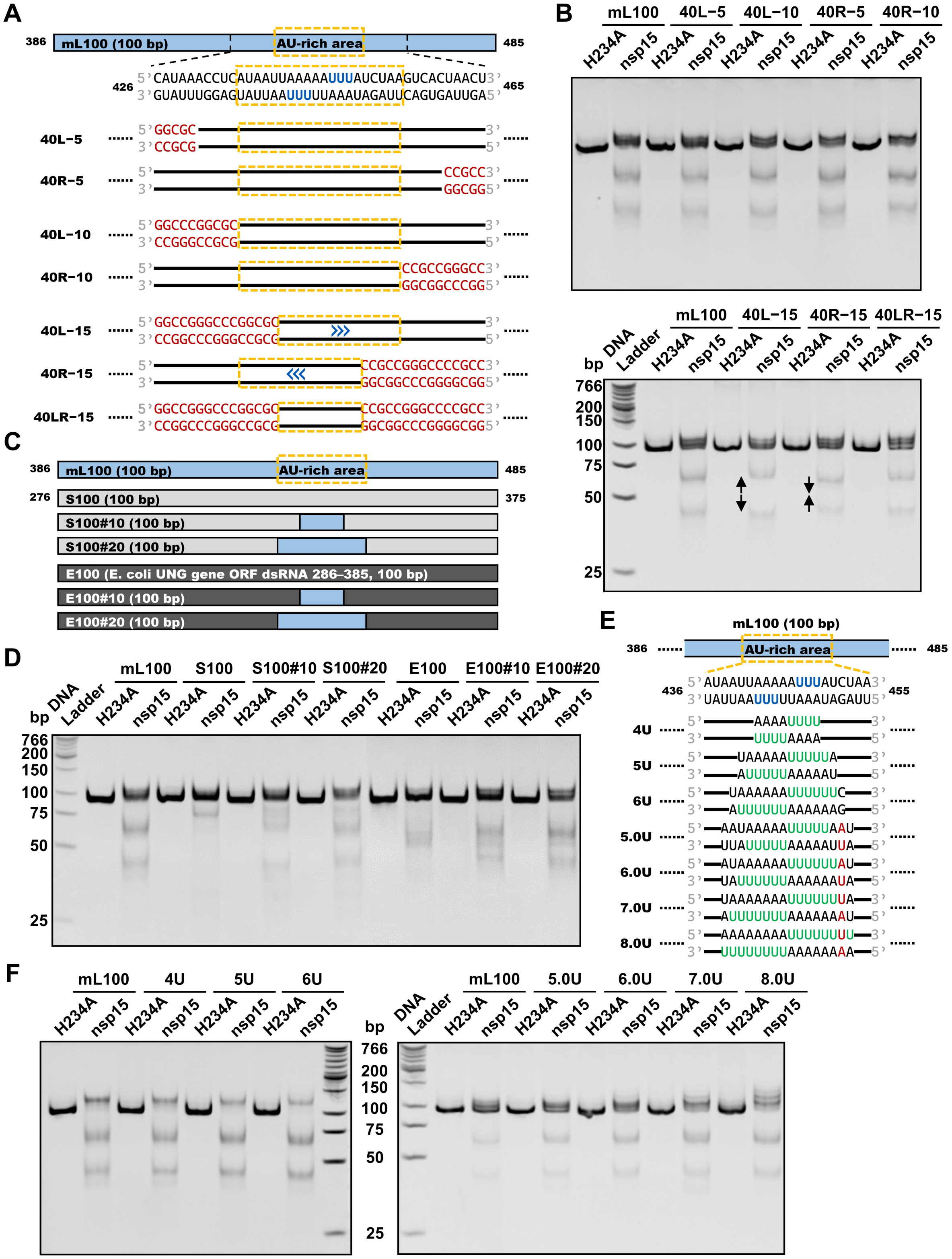

A
mL100 (100 bp)
AU-rich area
386
485
426
465
5' CAUAAACCUCAUAAUUAAAAAUUUAUCUAAGUCACUAACU 3'
3' GUAUUUGGAGUAUUAAUUUUUAAAUAGAUUCAGUGAUUGA 5'
40L-5
40R-5
40L-10
40R-10
40L-15
40R-15
40LR-15
B
mL100
40L-5
40L-10
40R-5
40R-10
H234A
nsp15
DNA Ladder
40L-15
40R-15
40LR-15
bp
766
200
150
100
75
50
25
C
mL100 (100 bp)
S100 (100 bp)
S100#10 (100 bp)
S100#20 (100 bp)
E100 (E. coli UNG gene ORF dsRNA 286–385, 100 bp)
E100#10 (100 bp)
E100#20 (100 bp)
276
375
D
S100
S100#10
S100#20
E100
E100#10
E100#20
E
436
455
5' AUAAUUAAAAAUUUAUCUAA 3'
3' UAUUAAUUUUUAAAUAGAUU 5'
4U
5U
6U
5.0U
6.0U
7.0U
8.0U
F

Figure S3. dsRNA cleavage by SARS-CoV-2 nsp15 is sensitive to AU arrangement (related to Figure 3).

(**A**) Schematic representation of the mL100 substrate and its variants used in (**B**). The three U sites with the strongest cleavage on every strand of the mL100 substrate identified previously are shown in blue. The AU-rich areas containing nsp15 preferred cleavage sites are marked with orange dashed boxes. The GC-rich sequences replacing the original sequences are shown in red. The black lines represent the original sequences. The blue arrows indicate the shift direction of the dsRNA cleavage sites in the variants as compared with the mL100 substrate. (**B**) AU-rich sequences flanking the cleavage sites facilitated cleavage by nsp15, while GC substitutions in the flanking sequences resulted in restricted cleavage nearby and a shift of the cleavage sites, as indicated by the size variations between the cleavage products from the mL100 substrate and its variants shown in (**A**). (**C**) Schematic representation of the S100 and E100 substrates and their variants used in (**D**). The sequence of the S100 substrate matched the 276–375-nucleotide sequence of the O967 substrate. The sequence of the E100 substrate matched the 286–385-nucleotide sequence of the *E. coli ung* gene ORF dsRNA substrate shown in **Figure 1E**. The variants contained 10-bp or 20-bp AU-rich sequences from the mL100 substrate. (**D**) The AU-rich sequences from the mL100 substrate conferred nsp15 cleavage on the S100 and E100 substrates. (**E**) Schematic representation of the mL100 substrate and its variants used in (**F**). Consecutive Us are shown in green. The AU or UA base pairs replacing the original CG base pairs are shown in red. (**F**) Consecutive Us enhance the cleavage by nsp15. The concentrations of nsp15 or H234A used for the left and right panel were 10 nM and 2.5 nM, respectively. All samples were analyzed by 12% TBE PAGE (native gel).

Figure S4

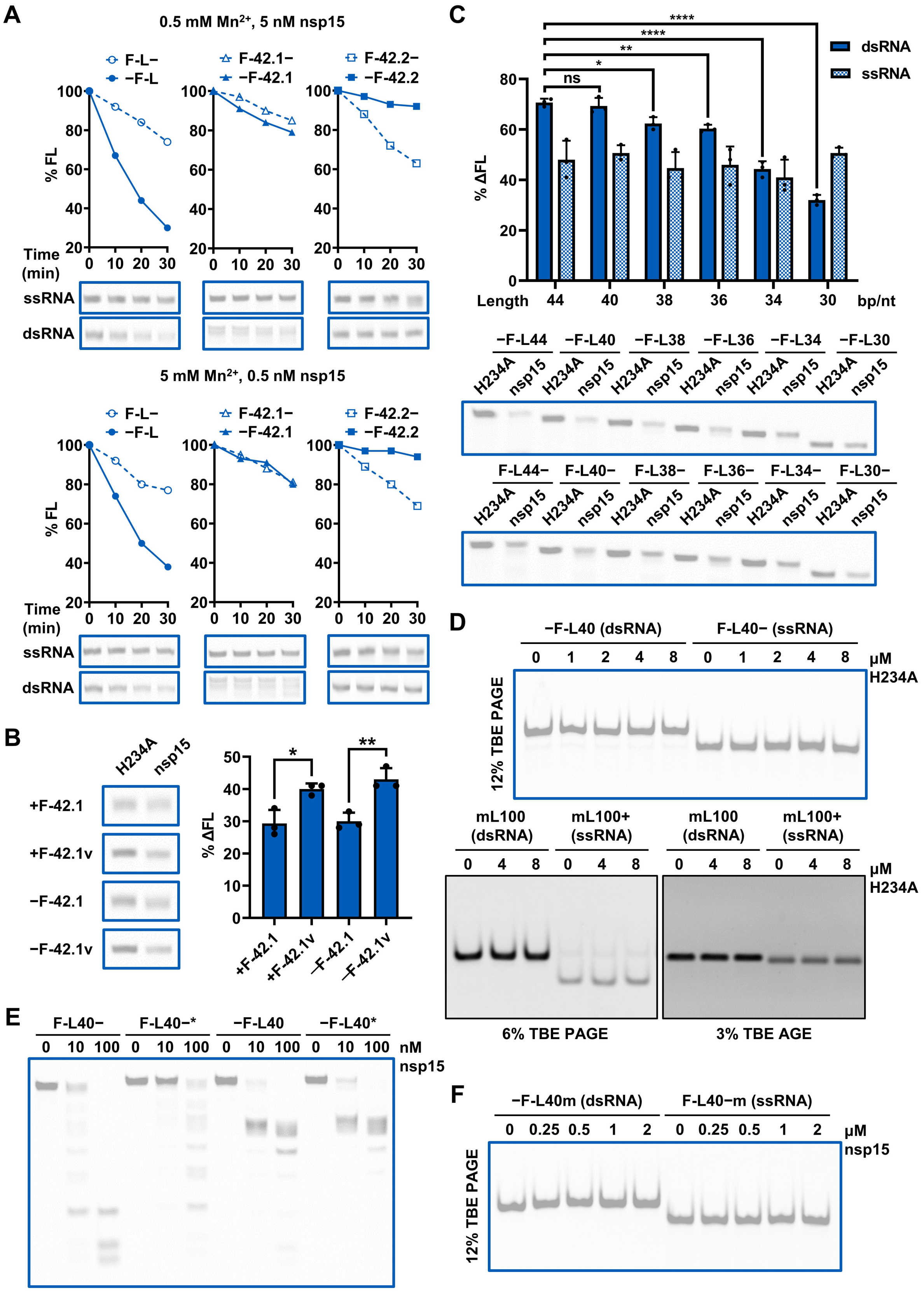

A
0.5 mM Mn²⁺, 5 nM nsp15
F-L−
−F-L
F-42.1−
−F-42.1
F-42.2−
−F-42.2
% FL
Time (min)
ssRNA
dsRNA
5 mM Mn²⁺, 0.5 nM nsp15
B
H234A
nsp15
+F-42.1
+F-42.1v
−F-42.1
−F-42.1v
% ΔFL
C
dsRNA
ssRNA
% ΔFL
Length
bp/nt
−F-L44
−F-L40
−F-L38
−F-L36
−F-L34
−F-L30
F-L44−
F-L40−
F-L38−
F-L36−
F-L34−
F-L30−
D
−F-L40 (dsRNA)
F-L40− (ssRNA)
μM H234A
12% TBE PAGE
mL100 (dsRNA)
mL100+ (ssRNA)
6% TBE PAGE
3% TBE AGE
E
F-L40−
F-L40−*
−F-L40
−F-L40*
nM nsp15
F
−F-L40m (dsRNA)
F-L40−m (ssRNA)
μM nsp15
12% TBE PAGE

Figure S4. Impact of AU content, AU distribution, and RNA length on nsp15 substrate preference (related to Figure 4).

(**A**) Cleavage of RNA substrates with various AU content and AU distributions by nsp15 in the presence of 0.5 mM or 5 mM Mn^{2+}. Remaining full-length RNA substrates after nsp15 cleavage were quantified as % FL. (**B**) Cleavage of the +F-42.1, +F-42.1v, −F-42.1, and −F-42.1v substrates showing the impact of a mismatch on nsp15 cleavage. (**C**) Cleavage of the variants of the −F-L substrate with various lengths and the corresponding ssRNA substrates by nsp15. In (**B and C**), reduction of the full-length substrate in every reaction was calculated as % ΔFL and the nsp15 H234A mutant was used as a negative control. The average and standard deviation of three independent reactions are graphed. Student's t-test was performed. ns, not significant, $p > 0.05$; *$p < 0.05$; **$p < 0.01$; ****$p < 0.0001$. (**D**) Binding of the nsp15 H234A mutant to dsRNA and ssRNA was detected by electrophoretic mobility shift assay. mL100+, the ssRNA substrate corresponding to the positive-sense strand of the mL100 substrate. (**E**) Cleavage of the F-L40− and −F-L40 substrates and their variants with phosphorothioate modification at the 3'-side of pyrimidines (designated as F-L40−* and −F-L40*) by various concentrations (0, 10, and 100 nM) of nsp15. (**F**) Binding of the wild-type nsp15 to the variants of the −F-L40 and F-L40− substrates with 2'-methoxy modification at all pyrimidine nucleotides (designated as −F-L40m and F-L40−m) was detected by electrophoretic mobility shift assay. All samples were analyzed by 20% TBE-urea PAGE (denaturing gel) unless otherwise indicated.

Figure S5

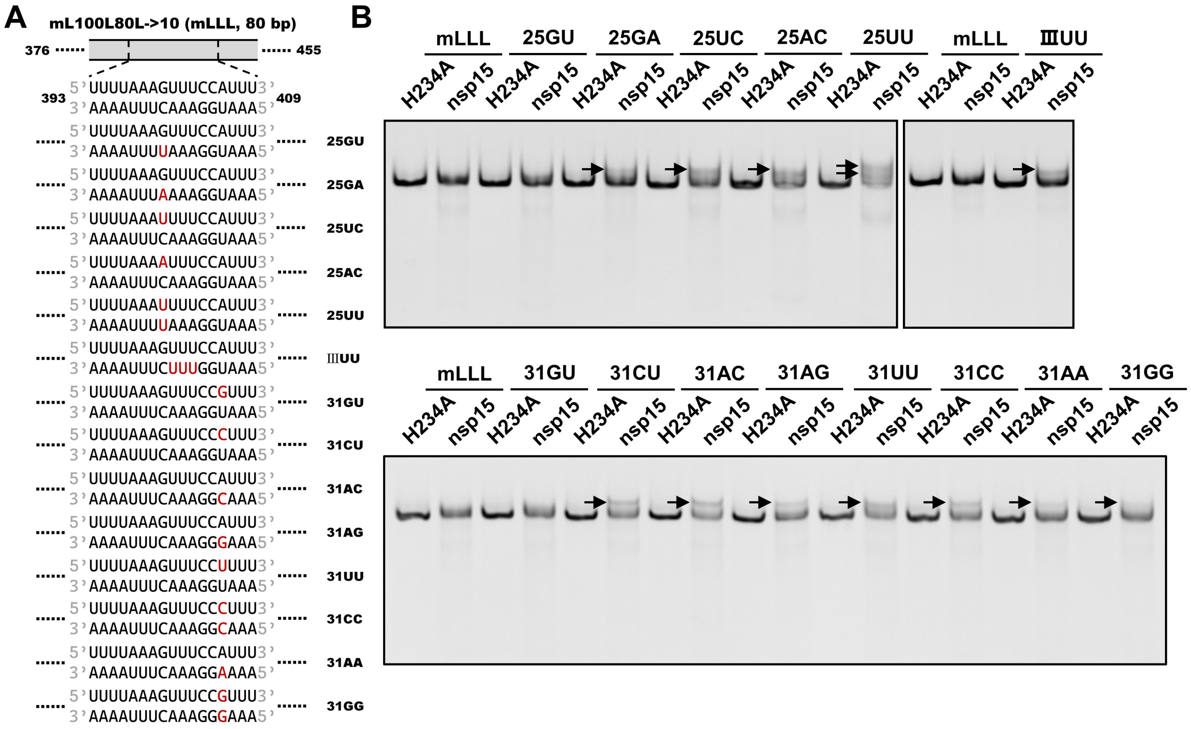

Figure S5. Cleavage of mismatch-containing dsRNA substrates by nsp15 (related to Figure 5).

(**A**) Schematic representation of the mL100L80L->10 substrate and its mismatch-containing variants. The bases replacing the original bases in the variants are shown in red. (**B**) Cleavage of the mL100L80L->10 substrate and its mismatch-containing variants shown in (**A**) by nsp15. Samples were analyzed by 12% TBE PAGE (native gel). The gel bands above the full-length substrate gel bands are marked by black arrows.

Figure S6A–E

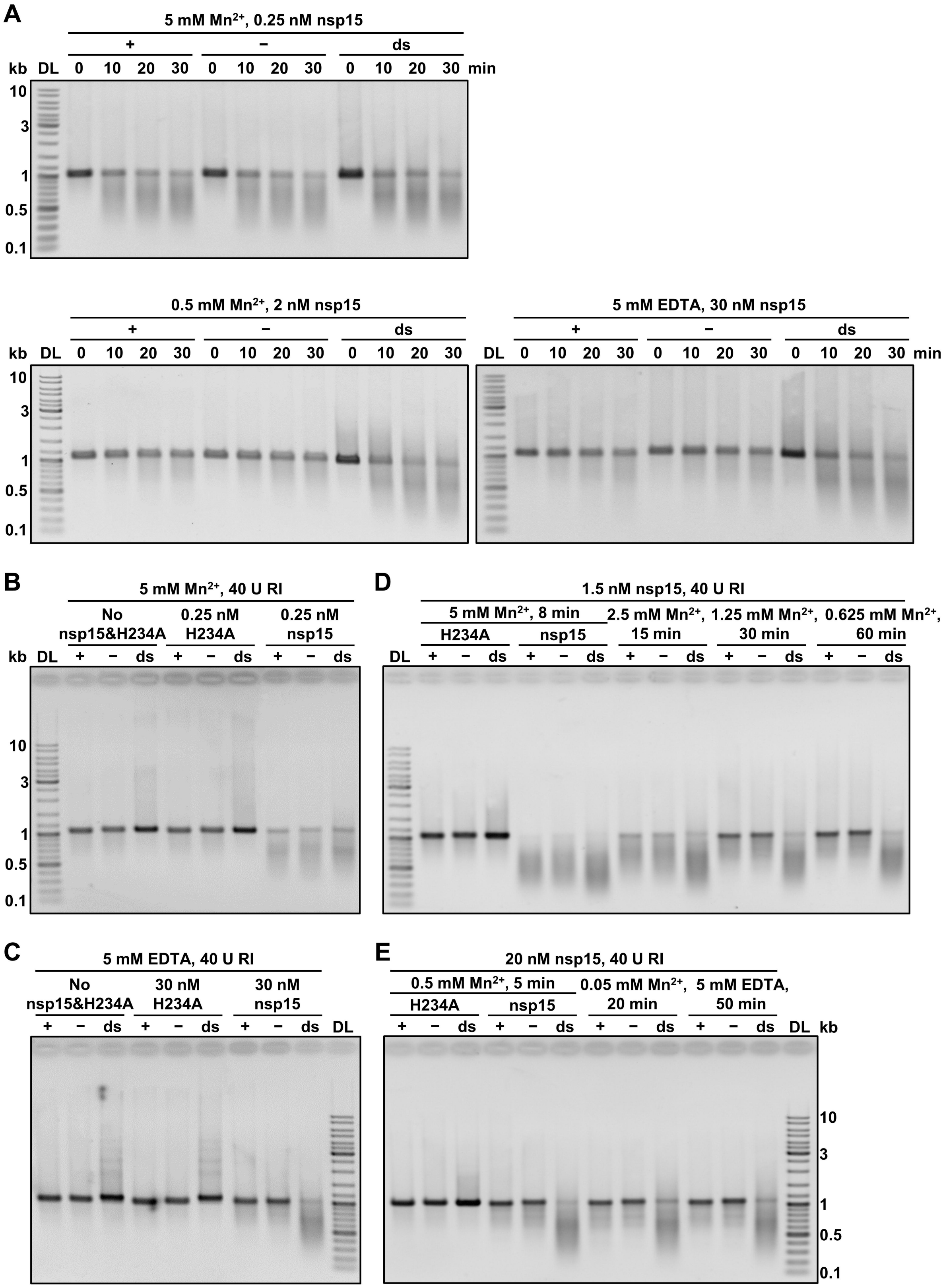
A
5 mM Mn2+, 0.25 nM nsp15
+
−
ds
kb DL 0 10 20 30 0 10 20 30 0 10 20 30 min
10
3
1
0.5
0.1
0.5 mM Mn2+, 2 nM nsp15
5 mM EDTA, 30 nM nsp15
+
−
ds
kb DL 0 10 20 30 0 10 20 30 0 10 20 30
DL 0 10 20 30 0 10 20 30 0 10 20 30 min
B
5 mM Mn2+, 40 U RI
No nsp15&H234A
0.25 nM H234A
0.25 nM nsp15
kb DL + − ds + − ds + − ds
D
1.5 nM nsp15, 40 U RI
5 mM Mn2+, 8 min
H234A
nsp15
2.5 mM Mn2+, 15 min
1.25 mM Mn2+, 30 min
0.625 mM Mn2+, 60 min
DL + − ds + − ds + − ds + − ds + − ds
C
5 mM EDTA, 40 U RI
No nsp15&H234A
30 nM H234A
30 nM nsp15
+ − ds + − ds + − ds DL
E
20 nM nsp15, 40 U RI
0.5 mM Mn2+, 5 min
H234A
nsp15
0.05 mM Mn2+, 20 min
5 mM EDTA, 50 min
+ − ds + − ds + − ds + − ds DL kb

Figure S6F

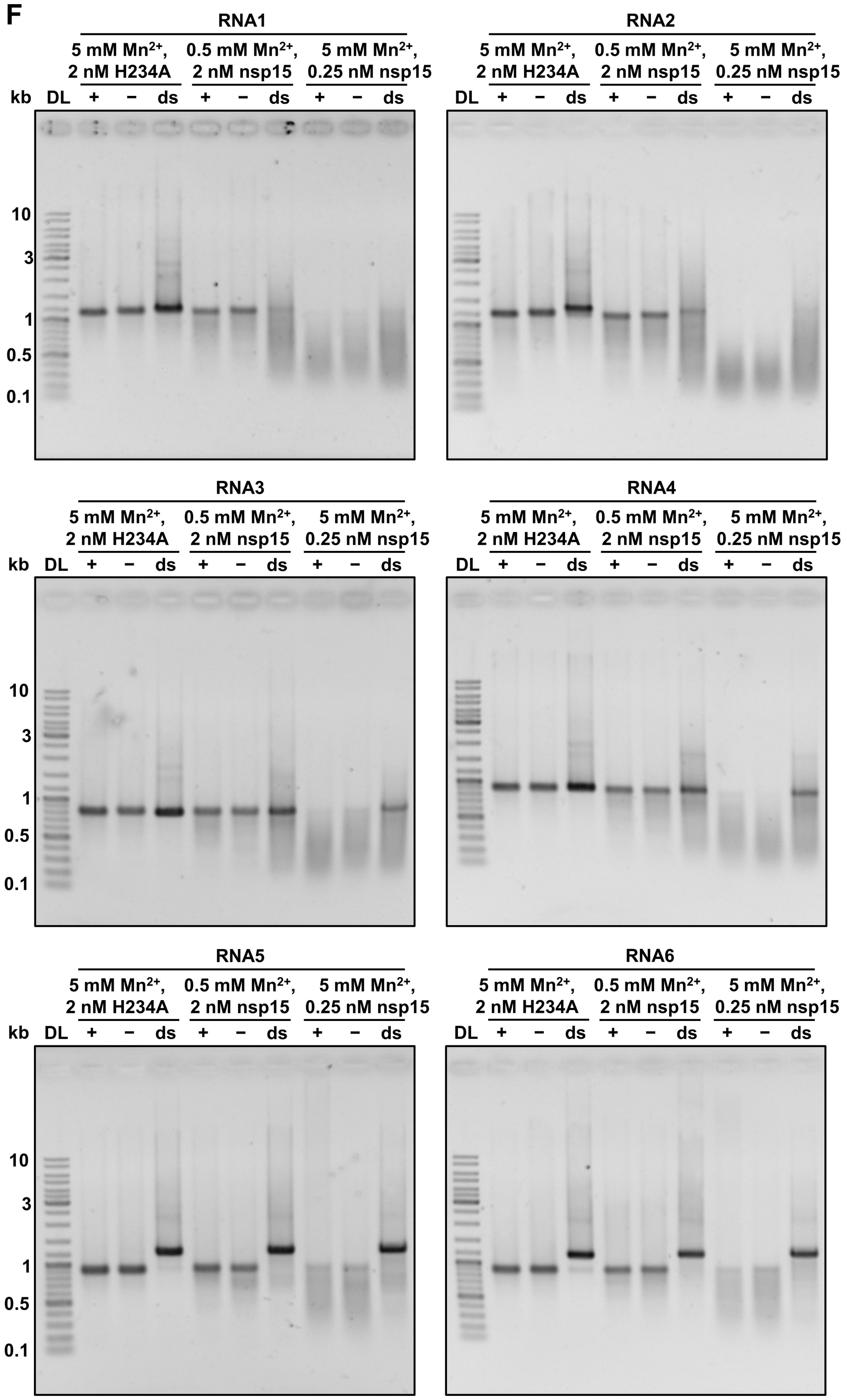

F
RNA1
RNA2
RNA3
RNA4
RNA5
RNA6
5 mM Mn^{2+}, 2 nM H234A
0.5 mM Mn^{2+}, 2 nM nsp15
5 mM Mn^{2+}, 0.25 nM nsp15
kb
DL
+
−
ds
10
3
1
0.5
0.1

Figure S6G

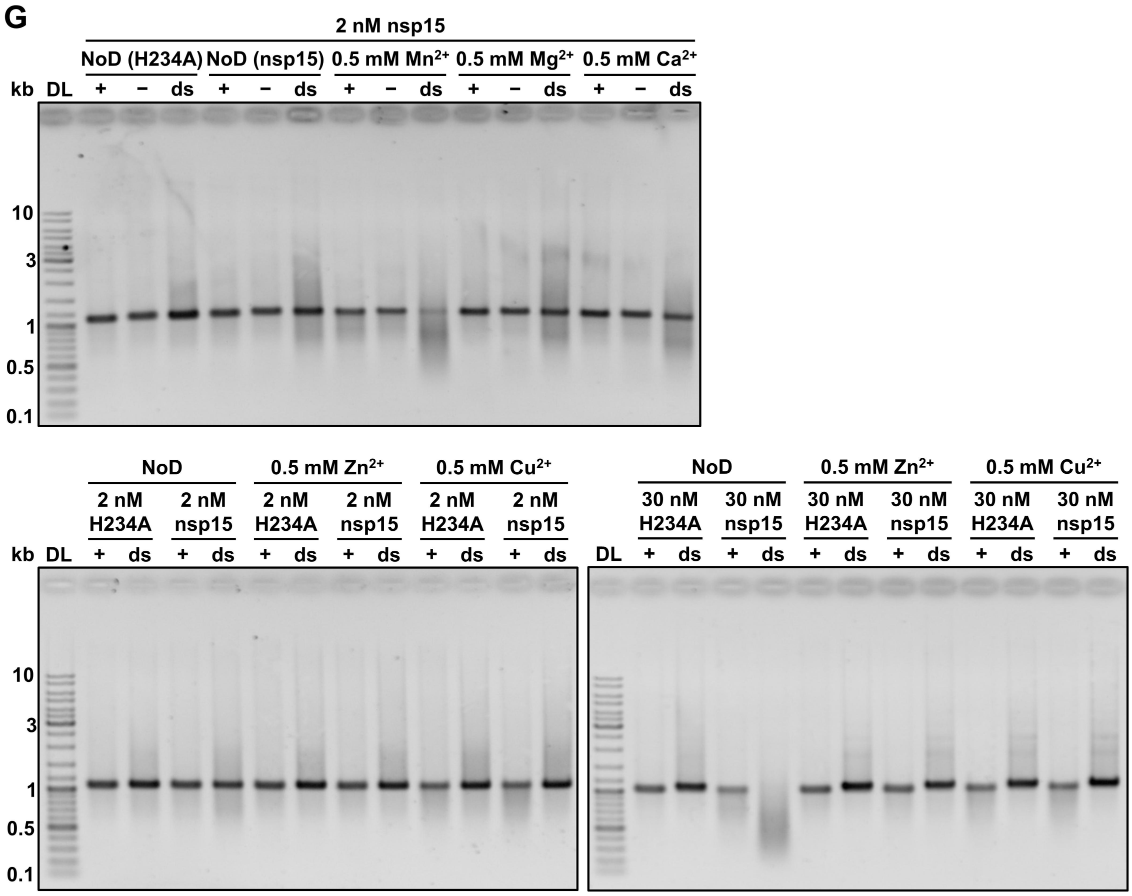

Figure S6. Full gel images corresponding to the quantitative results shown in Figure 1 and Supplementary Figure S1.

(**A**) Full gel images corresponding to **Figure 1B**. (**B**) Full gel image corresponding to **Supplementary Figure S1C**. (**C**) Full gel image corresponding to **Supplementary Figure S1D**. (**D**) Full gel image corresponding to **Supplementary Figure S1E**. (**E**) Full gel image corresponding to **Supplementary Figure S1F**. (**F**) Full gel images corresponding to **Figure 1F and Supplementary Figure S1G**. (**G**) Full gel images corresponding to **Supplementary Figure S1I**. DL refers to DNA ladder.
